# LENS: A New Pulsed Neutron Source for Research and Education

**DOI:** 10.6028/jres.110.016

**Published:** 2005-06-01

**Authors:** M. Leuschner, D. V. Baxter, J. M. Cameron, V. Derenchuk, C. Lavelle, A. Lone, H. Nann, T. Rinckel, W. M. Snow

**Affiliations:** Indiana University Cyclotron Facility, 2401 Milo B. Sampson Lane, Bloomington, IN 47408

**Keywords:** cold neutrons, neutron sources

## Abstract

A new pulsed neutron source is under construction at the Indiana University Cyclotron Facility (IUCF). Neutrons are produced via (p,n) reactions by a low-energy proton beam incident on a thin beryllium target. The source is tightly coupled to a cold methane moderator held at a temperature of 20 K or below. The resulting time-averaged cold neutron flux is expected to be comparable to that of the Intense Pulsed Neutron Source (IPNS) facility at Argonne National Laboratory. The initial experimental suite will include instrumentation for small angle neutron scattering (SANS), moderator studies, radiography, and zero-field spin-echo SANS.

## 1. Introduction

The Low Energy Neutron Source (LENS), currently under construction at the Indiana University Cyclotron Facility (IUCF), will be the world’s first university-based pulsed cold neutron source when it becomes operational in the spring of 2005. When completed, LENS will consist of a 13 MeV proton linac capable of producing a time-average current of 2.5 mA. The protons will be incident upon a beryllium target with a thickness of 3 mm, sufficient to stop all of the protons. The heat load on the beryllium target will be approximately 30 kW.

In contrast to other pulsed neutron sources which produce neutrons through spallation using high-energy proton beams, LENS will produce neutrons via low-energy (p,n) reactions. Although the neutron yield from (p,n) reactions is small compared to what is possible from spallation reactions, there are several compensating factors that combine to make LENS a viable facility for research and development. Since the limiting factor on proton beam intensity is often the ability to cool the target system, the time-averaged current can be much higher with a lower beam energy for a given power-on-target. The threshold for the (p,n) reaction is about 1.8 MeV, so the maximum neutron energy at LENS will only be 11.2 MeV. Starting with such a low energy production spectrum makes the moderation process easier and results in a relatively high cold neutron flux. Finally, the low heat load on the moderator allows for a favorable geometric coupling to the source. MCNP simulations suggest that the time-averaged LENS neutron intensity will be comparable to that of the IPNS facility at Argonne.

The neutron yield increases with increased proton energy. The proton energy for LENS was chosen to be as high as possible without introducing target activation problems. The 13.4 MeV threshold for the ^9^Be(p,t) reaction was therefore the limiting factor in the choice of the proton beam energy. The ^9^Be(p,t) reaction produces ^7^Be with a half-life of 53.2 d, as well as tritium with a half-life of 12.3 years.

The primary LENS moderator will be a (12 × 12) cm^2^ methane slab with a thickness of 1 cm. The combined neutron, gamma, and beta decay heat load on the methane moderator is expected to be on the order of 1 W when the facility is operated at its full power of 30 kW. Under these conditions we expect to be able to operate the moderator at temperatures well below 10 K, perhaps down to 4 K.

The combined target/moderator/reflector (TMR) assembly for LENS will be about 2 m in diameter. The choice of moderator and reflector materials has been carefully optimized to provide the greatest number of cold neutrons without sacrificing good timing characteristics. The reflector immediately surrounding the moderator will be liquid water. Although simulations showed that heavy water was superior in terms of producing a greater overall cold neutron flux, it also produced the undesirable effect of lengthening the time-emission spectrum considerably. Other possibilities considered for the reflector material were beryllium, carbon, and lead. Beryllium was the only candidate that performed as well as water, but the potential gains in flux were outweighed by the hazards associated with handling it. Replacing the water with beryllium remains an upgrade path for the future if it is warranted.

Immediately outside of the water reflector there will be a thin layer of borated poly whose function is to reduce the coupling of thermal neutrons to the outside environment. This decoupling serves to improve the time emission characteristics of the system. The remaining portion of the TMR assembly will be composed of alternating layers of lead and poly. The principal function of these layers will be to provide radiation shielding.

One of the guiding principles in the design of LENS was to preserve the capability for rapid prototyping of moderator systems. Thus it was deemed necessary to be able to safely access and modify components in the interior of the TMR assembly on timescales of a few days. This goal was accomplished by two means. First, the mechanical design of the TMR assembly includes a rail system so that a large sector of the TMR can be easily rolled away to expose the target and moderator core. Second, particular attention was paid to minimizing the presence of materials that activate with lifetimes greater than a few hours, thereby allowing personnel to safely access the exposed moderator shortly after the proton beam is turned off. The principal source of activation on this timescale is the decay of ^24^Na produced by the ^27^Al(n,α) reaction. ^24^Na has a lifetime of 15 h and emits more than 4 MeV of gamma rays when it decays.

While the time-averaged neutron flux at LENS is expected to be comparable to that of the IPNS, the gamma background is expected to be relatively low. The gamma background has two main components. The first component is due to the “prompt” (during the proton pulse) capture of neutrons in the borated poly decoupler or in the water reflector. More than 90 % of the primary neutrons from the beryllium target are captured in the water reflector and lost—resulting in the emission of a 2.2 MeV gamma ray. The second component arises from neutron capture on aluminum structural materials in the core of the TMR assembly. The ^27^Al(n,γ) reaction produces ^28^Al with a lifetime of 2.2 min. When it decays, it emits a 1.78 MeV gamma ray. This source of gamma rays is more of a problem at the instruments. The ^28^Al population reaches equilibrium shortly after beam is turned on and the decays produce a CW gamma flux in each of the neutron beamlines. The prompt gamma flux is not as important since it arrives at the instruments prior to the cold neutrons of interest and can therefore be discriminated against using time-of-flight methods.

The initial suite of instruments will occupy three beamlines. One beamline will house a conventional SANS instrument. A second beamline will be used for moderator studies, radiography, and neutron detector development. The third beamline will be dedicated to instrumentation development. The first instrument developed on this beamline will be a zero-field spin-echo spectrometer.

Perhaps the greatest impact the LENS facility will have on the field of neutron science will be in the area of moderator studies. The TMR assembly is designed specifically to facilitate moderator prototyping, as outlined previously. We plan to study new moderator materials and geometry, as well as the effect of other parameters such as pressure and temperature. LENS offers unparalleled flexibility in the control of the proton beam—the frequency, pulse width, and intensity can all be easily varied to suite the needs of the experimenter. Finally, due to the low heat load on the moderator, lower temperatures will be attainable. If we are able to shift the peak of the cold neutron flux down to the ≈1 meV range, then the development of ultra-cold neutron sources based on solid oxygen or superfluid ^4^He could be optimized. The lower radiation environment will allow LENS users to experiment with more “delicate” moderator materials that suffer radiation damage in spallation environments. Exploration of these lower moderator temperatures range required for ultra-cold neutron source development will be of great interest. Methane, for instance, undergoes a phase transition at 20 K. Its performance as a moderator in that regime has not been characterized.

The mission of the LENS facility is threefold. First, large amounts of beam time will be made available for training and education in the neutron sciences. Second, LENS will carry out a materials science research program centered on the SANS instrument. Efforts are underway to extend research on this instrument into nontraditional areas such as Biology and Chemistry. Finally, LENS will serve as a testbed for new source and instrumentation development efforts. Each of these three focuses will be coordinated to meet the future needs of larger facilities such as the Spallation Neutron Source, both in terms of expanding and nurturing the user community, and by developing technologies to maximize the scientific output.

